# A Hierarchical Three-Stage Feature Selection Strategy for Efficient and Generalizable sEMG-Based Joint Torque Estimation in Athletic Training and Rehabilitation

**DOI:** 10.3390/s26185768

**Published:** 2026-09-11

**Authors:** Yufeng Zheng, Pingao Huang, Hongqiang Wang, Yongxue Wang, Chunlong Gan, Hui Wang

**Affiliations:** 1 The Shenzhen Institutes of Advanced Technology, Chinese Academy of Sciences, Shenzhen 518055, China; yf.zheng2@siat.ac.cn; 2 The School of Electronic Engineering and Automation, Guilin University of Electronic Technology, Guilin 541004, China; huangpingao@126.com (P.H.); o1149385148@163.com (H.W.); 3 The College of Integrated Circuits, Shenzhen Polytechnic University, Shenzhen 518055, China; wangyongxue@szpu.edu.cn; 4 The Guizhou Institute of Sport Science (Guizhou Anti-Doping Agency), Guiyang 550025, China

**Keywords:** joint torque estimation, EMG, neuromuscular, feature extraction, regression analysis

## Abstract

**Highlights:**

**What are the main findings?**

- A hierarchical three-stage feature selection (H3FS) strategy was proposed to identify a compact and physiologically meaningful sEMG feature subset for joint torque estimation.
- The XGBoost model combined with the ELATE-3 feature set (LMAV, WL, and TKEO) achieved accurate and efficient torque prediction across shoulder, elbow, and knee joints with strong generalization across different athletic cohorts.

**What are the implications of the main findings?**

- The proposed framework provides a lightweight and interpretable alternative to computationally intensive approaches for real-time sEMG-based joint torque estimation.
- The H3FS strategy has potential applications in rehabilitation monitoring, human–machine interaction, and wearable neuromuscular assessment systems.

**Abstract:**

Estimating joint torque in athletes provides important information for understanding neuromuscular performance and optimizing high-intensity training. While surface electromyography (sEMG) offers a non-invasive measure of muscle activation, existing methods often struggle to balance prediction accuracy, computational efficiency, and generalizability across different movement conditions. This study proposes a Hierarchical Three-Stage Feature Selection (H3FS) strategy for efficient and stable sEMG-based joint torque estimation. H3FS integrates domain knowledge, statistical and model-driven screening to generate compact, physiologically meaningful feature sets tailored to various application contexts. Using isokinetic movement data from the shoulder, elbow, and knee joints of 39 young athletes, H3FS identified the optimal configuration, ELATE-3, comprising logarithmic mean absolute value (LMAV), waveform length (WL), and Teager–Kaiser energy operator (TKEO). The study also introduces an Integrated Performance Index (IPI) to evaluate prediction accuracy and computational cost. Results indicate that XGBoost with ELATE-3 achieves the best balance between accuracy and real-time performance (average R^2^ = 0.85, RMSE = 16.2, IPI = 0.013). It outperforms both traditional and data-driven feature sets, demonstrating strong generalization and low latency across joints, making it highly suitable for low-latency estimation on the adopted computational platform. The proposed strategy offers a systematic, scalable solution for joint torque estimation, supporting athletic training optimization and neuromuscular assessment, with potential future applications in rehabilitation monitoring.

## 1. Introduction

Athletes undergoing high-intensity training or specialized rehabilitation are frequently exposed to cumulative fatigue and joint overload. To effectively manage these loads and mitigate injury risks, continuous monitoring of joint torque is essential [1]. Surface electromyography (sEMG) is a widely established tool for this assessment [2], providing a non-invasive, dynamic measure of muscle function [3]. Crucially, sEMG captures neuromuscular activation without restricting natural movement [4], providing real-time feedback on muscle excitation and fatigue to inform training adjustments [5].

Joint torque estimation extends these capabilities by quantifying individual muscle function and establishing a direct mapping between sEMG signals and joint torque [6]. Unlike traditional methods limited to action classification, torque estimation determines the magnitude of the applied effort [7]. This distinction enables precise resistance control and the safer execution of motor tasks [7]. However, ensuring reliability in realistic movement scenarios remains a critical hurdle [8]. Existing methods struggle to handle dynamic factors, such as rapid load changes, variable speeds, and inter-subject variability. These limitations result in insufficient accuracy, latency, and poor generalization, creating significant barriers to the widespread deployment of torque estimation technologies in sports and rehabilitation [9].

Prediction accuracy is a primary metric for evaluating joint torque estimation [10]. Early research relied heavily on the Hill model, which analytically relates electrophysiological features to mechanical output [11]. While this approach is physiologically grounded, recent studies [12] highlight that its complex parameter identification process is highly sensitive to inter-subject variability and prone to overfitting, causing computational bottlenecks and performance fluctuations during dynamic tasks. To address these limitations, researchers have increasingly adopted data-driven nonlinear regression methods. For instance, recent comprehensive reviews [13] have demonstrated the superior efficacy of machine learning and deep neural networks in directly decoding complex, nonlinear neuromuscular patterns without the need for tedious biomechanical calibration. Ye et al. demonstrated that a LightGBM-based feature selection approach could reduce computational complexity without compromising accuracy, suggesting that lightweight models can be both efficient and precise [14]. Similarly, Hajian et al. achieved determination coefficients (R^2^) exceeding 0.94 during isometric contractions using a Bagged Tree Ensemble, further validating the potential of nonlinear models [15]. However, these successes are often limited to static or controlled settings, with performance degrading when movement, muscle, or signal variations occur. Furthermore, the lack of a unified standard for feature selection leads to inconsistent performance across different algorithms and models [16]. Consequently, developing a systematic strategy that ensures stable, high-accuracy predictions across diverse, complex conditions remains a critical challenge.

Real-time processing is a critical prerequisite for practical torque estimation. While deep learning architectures often achieve higher accuracy, their high computational costs often hinder real-time feasibility. For example, Su et al. reported inference latencies of 35–50 ms for a deep neural network [16]. Similarly, the physics-constrained approach by Ma et al. improves physiological consistency but significantly increases training overhead, pushing inference latency to the limits of real-time acceptability [17]. Furthermore, although Fialkoff et al. achieved high precision (R^2^ = 0.95) using EMG imaging, the system remains limited to offline validation, precluding its use for continuous online feedback [18].

Given the diversity of rehabilitation tasks and inherent physiological variability, model generalization is critical for practical deployment. Athletes exhibit significant differences in muscle architecture, neural control strategies, fatigue levels, and training intensities. Furthermore, motor tasks vary widely, ranging from isolated single-joint movements to complex multi-joint coordination. However, most existing models are trained and validated under fixed, single-joint conditions. Consequently, performance degrades sharply when applied to new subjects, movement patterns, or scenarios. For instance, Mokri et al. optimized a support vector regression model using a genetic algorithm, achieving an impressive R^2^ of 0.99 for a knee-joint task [19]. Yet, this accuracy proved highly specific to the training conditions, with performance dropping significantly when tested across different movements or subjects. Similarly, Saranya and Poonguzhali attempted to generalize feature sets using a three-layer selection method, but their validation was restricted to static isometric tasks, leaving dynamic and cross-joint scenarios unexplored [20]. Recent studies [21] have similarly emphasized the critical necessity of cross-joint transferability and dynamic evaluation to ensure the generalizability and robustness of sEMG-based torque estimation. These limitations underscore the need for torque estimation models to prioritize adaptability alongside accuracy. Developing a strategy that balances high precision, computational efficiency, and robust generalization across diverse subjects, joints, and dynamic conditions remains a fundamental objective.

Despite these advancements, a significant gap remains in systematic feature selection tailored to high-intensity, multi-joint athletic scenarios. Existing feature selection methods for joint torque estimation primarily rely on empirical subsets, such as the Hudgin set, or on computationally intensive wrapper-based methods, such as genetic algorithms. While these approaches can be effective for specific tasks, they often lack a unified decision criterion to balance predictive fidelity with the low-latency requirements of real-time rehabilitation. Furthermore, most existing feature sets are validated under single-joint or isometric conditions, failing to account for the complex neuromuscular recruitment patterns inherent to elite athletes across different sporting disciplines. Therefore, there is a critical need for a principled selection strategy that ensures both physiological interpretability and cross-joint robustness.

To address the critical trade-offs between prediction accuracy, computational efficiency, and generalization of joint torque estimation, this study proposes a Hierarchical Three-Stage Feature Selection (H3FS) strategy for sEMG analysis. By systematically integrating domain knowledge, statistical evaluation, and model-driven screening, H3FS identifies feature subsets that maximize predictive performance while minimizing computational overhead. The primary contributions of this work are threefold: (1) This study introduces the H3FS strategy, a principled protocol guided by the Integrated Performance Index (IPI), to systematically reconcile the trade-off between estimation accuracy and computational efficiency; (2) Unlike studies restricted to single-joint or static tasks, the ELATE-3 feature set is validated across three major joints (shoulder, elbow, and knee) and four distinct athletic cohorts (39 athletes), ensuring robust generalization in dynamic scenarios; (3) The results identify the XGBoost–ELATE-3 configuration as a “minimalist but high-fidelity” solution, achieving superior performance consistency and sub-millisecond latency across all tested joints and sports disciplines.

The proposed strategy in this paper underwent comprehensive performance tests, covering multiple aspects, including athletes from different sports, joints (shoulder, elbow, and knee), and comparisons with various benchmark regression algorithms. The performance of the proposed strategy was evaluated.

## 2. Materials and Methods

### 2.1. Participants and Experimental Protocol

A total of 39 healthy young athletes with no history of neurological or musculoskeletal disorders participated in this study. The participants were recruited from four specialized athletic teams: archery (*n* = 9), wrestling (*n* = 8), rock climbing (*n* = 11), and Taekwondo (*n* = 11).

The experimental protocol involved isokinetic torque assessments of the dominant shoulder, elbow, and knee joints. Before data collection, all subjects provided written informed consent as per the procedures approved by the institutional ethics review committee (Ethics Approval No.: SIAT-IRB-240115-H0708).

**Figure 1:**
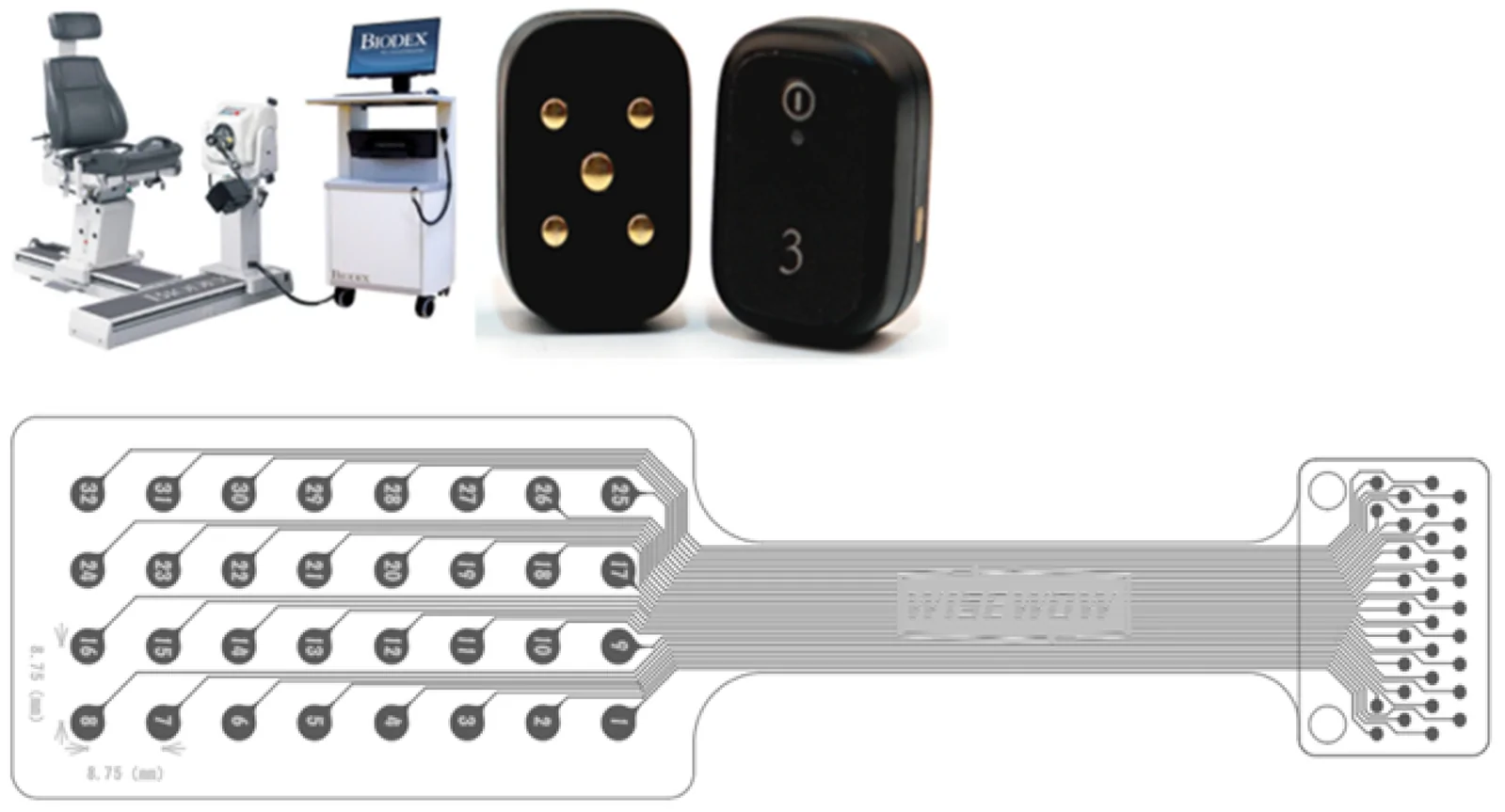
Experimental setup and data acquisition hardware. The system integrates a Biodex isokinetic dynamometer for precise torque measurement. Muscle activation is captured via a wireless sEMG sensor node connected to a 32-channel flexible electrode array.

As shown in Figure 1, the experimental setup synchronized a multi-joint isokinetic dynamometer (Biodex System 4, Biodex Medical Systems, Shirley, NY, USA) with a wireless sEMG acquisition system (RT-R0016S, Shenzhen RiYiTaiYi Technology Co., Ltd., Shenzhen, China). The system featured compact wireless measurement units (16.1 g, 4000 Hz sampling, 24-bit resolution) and utilized a separate 32-channel flexible printed circuit (FPC) electrode array (8 × 4 rectangular grid layout) to capture high-resolution surface signals with optimal skin conformity.

Temporal synchronization between the sEMG and Biodex systems was achieved via a visual-bridged protocol. A central signal generator hardware-locked the sEMG devices and a high-speed camera to a unified timebase. The camera recorded the precise onset of the dynamometer’s lever movement, enabling offline alignment of the Biodex kinematic data with the sEMG signals using this visual reference. Torque was measured using the Biodex System 4 dynamometer (±1% dynamic accuracy), with standard gravitational corrections applied via its proprietary software prior to testing.

**Figure 2:**
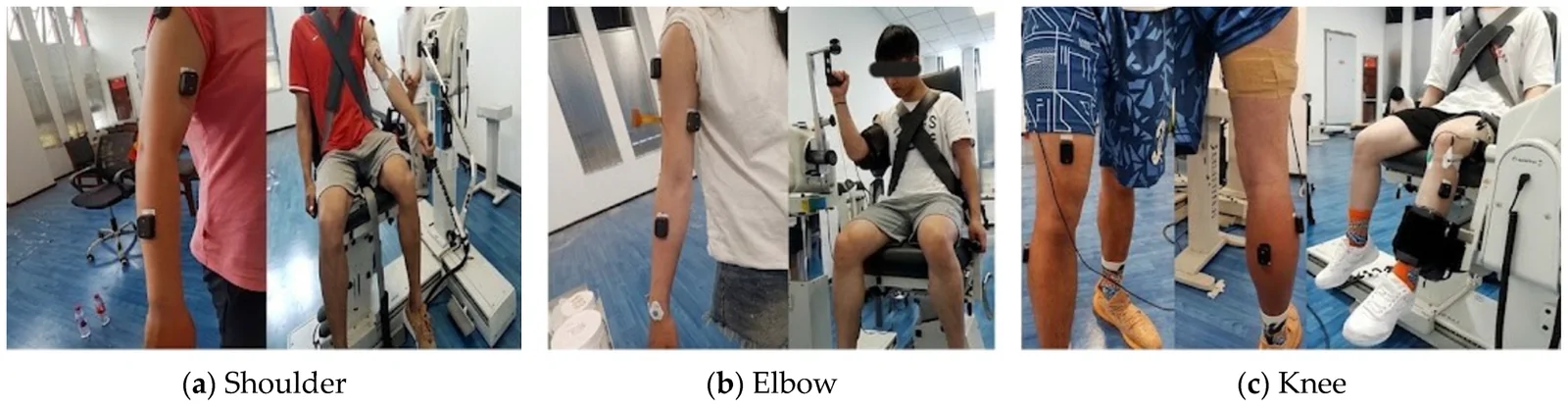
Experimental apparatus for isokinetic torque assessment. Illustrated are the specific multi-joint devices and integrated multi-sensor arrays used in the protocol.

Following standard skin preparation and familiarization trials, isokinetic torque was assessed on the dominant shoulder, elbow, and knee joints (Figure 2, Table 1). The protocol encompassed three angular velocities (45°/s, 90°/s, 120°/s), each comprising three repetitions with a 120-s inter-set rest to prevent fatigue.

Specifically, the experimental design yielded 351 distinct trial blocks (39 subjects × 3 joints × 3 velocities). A single trial block comprised three consecutive repetitions. When accounting for pure isokinetic execution, mechanical cushioning, and necessary directional transition pauses, each continuous recording block generated approximately 300 to 1500 time-series windows (using a 50 ms stride), depending on the joint’s range of motion and angular velocity. This structural diversity confirms the observational independence of the recordings, ultimately aggregating to a robust total of 320,837 valid windows across the entire cohort.

### 2.2. Data Pre-Processing and Feature Extraction

In this study, sEMG signals were acquired at 2000 Hz, while kinematic variables (angular velocity and torque) were recorded at 100 Hz. To ensure precise temporal alignment, the 100 Hz kinematic data were upsampled to 2000 Hz using a polyphase FIR antialiasing filter, which preserves low-frequency dynamics while preventing high-frequency artifacts. Signal preprocessing was implemented via the following pipeline:

1. Spatial Averaging: To mitigate artifacts caused by electrode edge lifting, analysis was restricted to centrally located channels (10–15 and 18–23; see Figure 1). Signals from these channels were averaged to derive a robust, representative muscle activation profile.
2. Band-pass Filtering: A fourth-order Butterworth filter (20–400 Hz) was applied to isolate the physiological bandwidth.
3. Artifact Removal: Powerline interference was suppressed using a second-order IIR notch filter targeting 50 Hz and its harmonics (100 Hz, 150 Hz, 200 Hz).
4. Envelope Extraction: Filtered signals were full-wave rectified and smoothed via a fourth-order low-pass Butterworth filter with a 3 Hz cutoff frequency.
5. Normalization: To address inter-channel variability, the envelope of each channel was normalized independently prior to dataset partitioning. The normalized signal $x\prime_{i}\left(t\right)$ for channel i was defined as $x\prime_{i}\left(t\right)=x_{i}\left(t\right)/\left(\max\left(x_{i}\right)+\epsilon \right)$, where ϵ is a small constant. Crucially, the peak amplitude $\max\left(x_{i}\right)$ was computed strictly on a per-trial basis rather than globally across the cohort.

**Figure 3:**
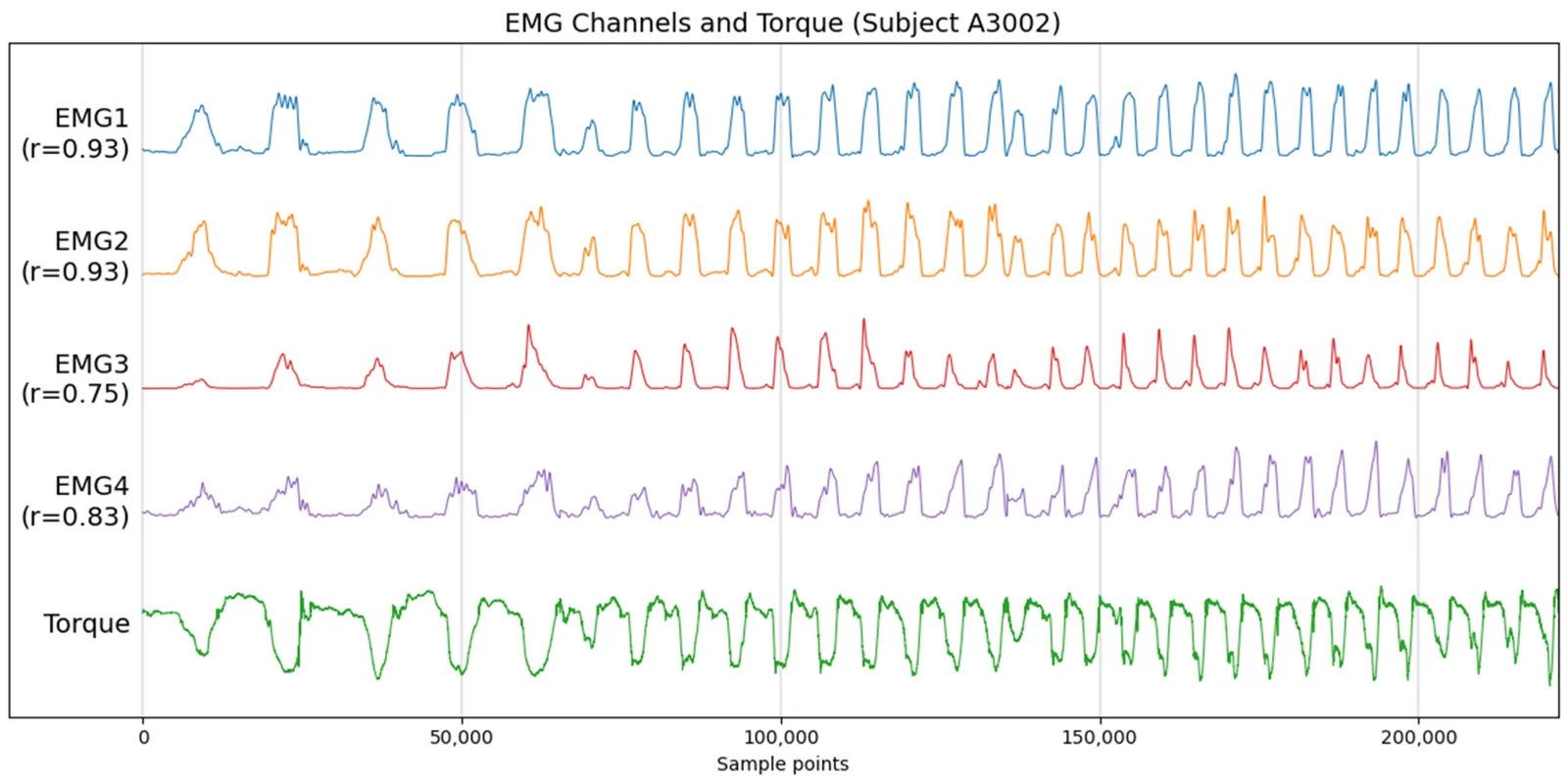
Knee joint EMG channels and torque from subject A3002.

Figure 3 shows the timing link between four sEMG channels and the torque profile. Pearson correlation confirmed a strong positive link between sEMG amplitude and torque output.

To capture the temporal and spectral dynamics of muscle activation, we extracted a comprehensive set of features spanning the time [22], frequency [23], and time–frequency domains [24]. Table 2 shows 21 metrics per input channel. The feature vector combines three sensor channels and one from the spatially averaged array, totaling 84 features (21 × 4). Extraction used a sliding window of 100 ms with 50 ms stride (50% overlap). Notably, explicit compensation for electromechanical delay (EMD) was omitted, as the 100 ms window naturally covers the typical EMD range (30–100 ms), enabling the models to implicitly learn this delayed mapping.

### 2.3. Feature Selection

**Figure 4:**
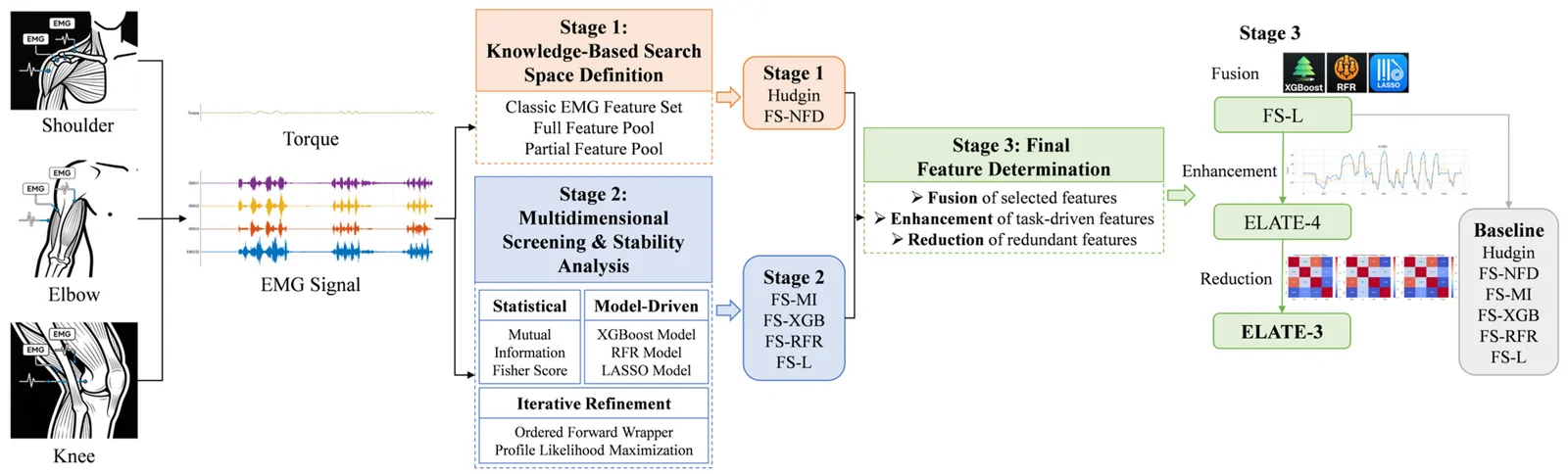
Diagram of the proposed H3FS pipeline and the resulting feature sets at each selection stage.

Effective feature selection is a prerequisite for robust predictive modeling. To construct a generalized feature set specialized for joint torque estimation, we propose the Hierarchical Three-Stage Feature Selection (H3FS) strategy (Figure 4). This architecture integrates domain knowledge, statistical evaluation, and model-driven screening to identify high-value feature subsets. To maintain a principled, consistent decision-making process across all stages, we used the Integrated Performance Index (IPI) as the core selection criterion. Briefly, the IPI is a weighted composite metric ($\mathrm{IPI}=\alpha \cdot e_{c}+\beta \cdot (1-d_{c})+\gamma \cdot t_{c}$) that quantifies the trade-off between prediction accuracy and computational efficiency (see Section 2.5 for the formal definition). By utilizing the IPI as a steering metric, the H3FS strategy ensures adaptability across diverse neuromuscular contexts while strictly limiting system latency. The entire H3FS workflow was conducted strictly on the training set, while the independent test set was isolated and used exclusively for final model benchmarking.

#### 2.3.1. Stage 1: Knowledge-Based Search Space Definition

Stage 1 establishes an interpretable baseline using classical EMG features with proven physiological relevance, specifically the widely adopted Hudgin feature set [25]. Concurrently, we constructed an extended candidate pool encompassing a broad spectrum of time-domain, frequency-domain, and nonlinear complexity metrics. The core objective of this stage is to define a candidate space that satisfies the prerequisite of physiological interpretability while ensuring that each baseline candidate maintains a favorable initial IPI, balancing raw predictive potential with low computational complexity.

#### 2.3.2. Stage 2: Multidimensional Screening and Stability Analysis

Stage 2 filters the extended feature pool to isolate high-relevance, low-redundancy predictors using a hybrid approach. This process involves three distinct decision rules guided by the IPI to evaluate the marginal utility of each feature. (1) Statistical methods, including mutual information [26] and Fisher score [27], were used to quantify the correlation with joint torque and the discriminative ability of each feature. To ensure cross-scenario stability, we identified the intersection of top-scoring features across different joint conditions (shoulder, elbow, and knee), retaining only those with consistent IPI performance across tasks. (2) Model-driven methods, such as Least Absolute Shrinkage and Selection Operator (LASSO) regression with L1 regularization [28] or the eXtreme Gradient Boosting model (XGBoost) [29], were used to rank features based on their intrinsic contribution to torque prediction. By sorting and selecting the top-ranked features based on the built-in importance scores, each model-driven method generated its corresponding model feature set. (3) Iterative refinement strategies (such as Ordered Forward Wrapping [30] and Profile Likelihood Maximization [31]) were further applied to identify the parsimonious subset. In this step, the IPI served as the objective threshold to determine whether including an additional feature yielded sufficient accuracy gains to justify its incremental computational cost.

#### 2.3.3. Stage 3: Final Feature Determination

Stage 3 synthesizes the candidate pools from the previous stages to construct a final subset that balances interpretability, compactness, and predictive fidelity. In this stage, the IPI serves as the ultimate criterion for evaluating the benefit-to-cost ratio of candidate sets. To maximize generalizability and identify the most suitable regression architecture, we conducted a multi-model comparative evaluation across XGBoost, Random Forest Regression (RFR), and LASSO. Specifically, the evaluation followed a straightforward sequence: the candidate feature subsets were first utilized to train these three distinct models exclusively on the training set. Subsequently, their relative predictive performances were systematically benchmarked to finalize the most suitable feature-model configuration strictly prior to independent evaluation on the hold-out test set.

Applying the H3FS strategy to the current dataset yielded the ELATE (EMG LASSO and Teager–Kaiser Enhanced) configuration. Comparative validation in Stage 2 identified the triad of RMS, LMAV, and WL as the high-performing core. To capture transient energy fluctuations essential for dynamic tasks [32], the Teager–Kaiser Energy Operator (TKEO) was integrated, yielding an intermediate four-feature set (ELATE-4). However, subsequent multicollinearity analysis revealed strong collinearity between RMS and LMAV, prompting pruning of RMS to improve computational efficiency. The final ELATE-3 configuration (LMAV, WL, TKEO) offers a compact, interpretable, robust feature vector for joint torque estimation.

### 2.4. Regression Model

To comprehensively evaluate feature set performance, we employed three distinct regression algorithms representing different modeling paradigms: (1) XGBoost, a tree-based gradient boosting model that captures nonlinear relationships and provides robust generalization even under default hyperparameter settings [29]; (2) RFR, a classic Bagging-based ensemble model employed to examine stability under feature perturbation and cross-scenario conditions [33]; and (3) LASSO, a linear model with L1 regularization that yields sparse and interpretable baseline predictions [28].

For a given input feature vector $x\in R^{d}$ (where d is the dimensionality of the selected feature set), the goal is to estimate the joint torque $\hat{\tau }$ through a mapping function f(x):

(1) $$\hat{\tau }=f\left(x\right)+\epsilon$$

where ϵ represents the residual error. Among the three models, XGBoost served as the primary architecture. Its core principle is to approximate the target function using an ensemble of K additive trees:

(2) $${\hat{\tau }}_{i}=\sum_{k=1}^{K}f_{k}\left(x_{i}\right),f_{k}\in F$$

where F is the space of regression trees. The model parameters are learned by minimizing the following regularized objective function:

(3) $$L=\sum_{i}L(\tau_{i},{\hat{\tau }}_{i})+\sum_{k}\Omega (f_{k})$$

The training process minimizes the Mean Squared Error (MSE) as the loss function L, while the complexity term Ω uses $L_{1}$ and $L_{2}$ regularization to prevent overfitting and ensure robust generalization.

To ensure that performance variations were attributable solely to feature quality rather than model-specific tuning, all algorithms were initialized with standardized hyperparameters: XGBoost (n_estimators = 20, max_depth = 6, learning_rate = 0.3), RFR (n_estimators = 300, max_depth = None), and LASSO (alpha = 0.01, max_iter = 100). This unified configuration isolates the intrinsic discriminative power of the feature sets, allowing for a systematic evaluation of the ELATE-3 configuration’s efficacy in joint torque estimation.

### 2.5. Evaluation Criteria

To evaluate the selected feature sets, three evaluation metrics were used:

1. Root Mean Square Error (RMSE) is a commonly used metric for quantifying the prediction error of joint torque estimation. It represents the square root of the mean of the squared differences between the observed and the predicted torque values and is defined as follows:

(4) $$RMSE=\sqrt{\frac{1}{n}\sum_{t=1}^{n}{\left({\hat{y}}_{t}-y_{t}\right)}^{2}}$$

where ${\hat{y}}_{t}$ and $y_{t}$ represent the predicted and the observed joint torque, respectively, and n is the total number of samples. A smaller RMSE indicates higher prediction accuracy.

To address the sensitivity of RMSE to the range of values, this study also used the Normalized Root Mean Square Error (NRMSE). NRMSE normalizes the RMSE by the range of the target variable and is defined as follows:

(5) $$NRMSE=\frac{\sqrt{\frac{1}{n}\sum_{t=1}^{n}{\left({\hat{y}}_{t}-y_{t}\right)}^{2}}}{y_{max}-y_{min}}$$

where $y_{max}$ and $y_{min}$ represent the maximum and minimum values of the observed data, respectively. A smaller NRMSE indicates better model performance and provides stronger generalization across different signal amplitudes and subjects.

2. Coefficient of Determination (R^2^) measures the proportion of variance in the observed data that is explained by the prediction model. It is defined as follows:

(6) $$R^{2}=1-\frac{\sum_{t=1}^{n}{\left(y_{t}-{\hat{y}}_{t}\right)}^{2}}{\sum_{t=1}^{n}{\left(y_{t}-\bar{y}\right)}^{2}}$$

where $\bar{y}$ represents the mean of the observed values, and an R^2^ value closer to 1 indicates that the model explains a higher proportion of the variance and has better prediction accuracy.

3. TimeSpent is adopted to evaluate computation time and assess the real-time feasibility of each model. The total time consists of two parts: feature extraction time and prediction time, as defined in Equation (7):

(7) $$\mathrm{TimeSpent}=T_{\mathrm{feature}}+T_{\mathrm{prediction}}$$

where $T_{\mathrm{feature}}$ represents the average time required to extract all features within a single analysis window, and $T_{\mathrm{prediction}}$ denotes the average inference time for one prediction cycle of the regression model. A smaller TimeSpent value indicates higher computational efficiency and stronger potential for real-time or online applications.

However, reliance on these metrics in isolation presents significant interpretability challenges. First, NRMSE and R^2^ operate on disparate scales, which can obscure direct comparisons between models. Second, computational cost (inference latency) is structurally independent of predictive fidelity; a higher computational burden is often the price of superior accuracy, yet raw latency metrics fail to capture this trade-off. Consequently, evaluating these factors separately is insufficient for optimizing real-time rehabilitation systems [34]. To address this trade-off, we introduced the Integrated Performance Index (IPI) as a pragmatic multi-objective decision-support index. While the assignment of weights (α,β,γ) inherently reflects a practical, application-driven engineering trade-off rather than an absolute physical law, our sensitivity analysis (Section 3.5) confirms that the resulting model hierarchy remains robust across a broad spectrum of weight configurations. As previously described, the IPI serves both as the internal selection criterion for the H3FS strategy and as a global performance score.

Prior to aggregation, normalization is essential to reconcile the disparate units and magnitudes of the constituent metrics. To achieve this, we applied min–max normalization across all evaluated feature-model configurations. This transformation maps NRMSE, R^2^, and inference latency to a standardized [0, 1] interval, facilitating dimensionless integration:

(8) $$\begin{array}{c} e_{c}=\frac{{\mathrm{NRMSE}}_{c}-\underset{j\in \Omega }{min}({\mathrm{NRMSE}}_{j})}{\underset{j\in \Omega }{max}({\mathrm{NRMSE}}_{j})-\underset{j\in \Omega }{min}({\mathrm{NRMSE}}_{j})+\varepsilon }\in \left[0,1\right]\\ d_{c}=\frac{R_{c}^{2}-\underset{j\in \Omega }{min}(R_{j}^{2})}{\underset{j\in \Omega }{max}(R_{j}^{2})-\underset{j\in \Omega }{min}(R_{j}^{2})+\varepsilon }\in \left[0,1\right]\\ t_{c}=\frac{{TimeSpent}_{c}-\underset{j\in \Omega }{min}({TimeSpent}_{j})}{\underset{j\in \Omega }{max}({TimeSpent}_{j})-\underset{j\in \Omega }{min}({TimeSpent}_{j})+\varepsilon }\in \left[0,1\right] \end{array}$$

where $e_{c}$, $d_{c}$, and $t_{c}$ represent the normalized NRMSE, R^2^, and TimeSpent values of model c, respectively. The term ε>0 (typically set to $10^{-12}$) is a small constant to prevent division by zero.

The Integrated Performance Index (IPI) is then defined as a weighted linear combination:

(9) $$\mathrm{IPI}=\alpha \cdot e_{c}+\beta \cdot (1-d_{c})+\gamma \cdot t_{c}$$

where α, β, and γ are non-negative weighting coefficients that satisfy α+β+γ=1. Based on empirical considerations, the weights in this study were set to *α* = 0.4, *β* = 0.4, and *γ* = 0.2 to balance model accuracy and computational efficiency.

The theoretical utility of the IPI lies in its unification of multi-dimensional performance criteria into a single scalar metric. While normalized NRMSE and R^2^ characterize predictive fidelity, the normalized latency term accounts for computational overhead. By rendering these distinct attributes dimensionless and scale-consistent, the IPI facilitates standardized intra-cohort comparisons across heterogeneous architectures. Consequently, a lower IPI reflects superior aggregate performance, indicating a favorable balance between prediction accuracy and operational efficiency.

The IPI was specifically designed as an internal heuristic to guide the H3FS feature selection process. For the final benchmarking on the independent test set, the ELATE-3 configuration was evaluated exclusively using standard metrics (RMSE, NRMSE, R^2^, and raw inference latency) to ensure a strictly objective assessment.

### 2.6. Baseline Methods

To ensure rigorous benchmarking, we evaluated the proposed H3FS strategy against a suite of representative baseline methods under identical experimental conditions. The comparative analysis included the classical Hudgin set, a composite non-frequency domain set, and four ranking-based strategies driven by the Mutual Information, XGBoost, RFR, and LASSO importance scores (detailed in Table 3). This selection encompasses diverse methodological paradigms, ranging from domain knowledge to statistical relevance and model-driven screening, providing a robust validation ground for the H3FS strategy and the derived ELATE-3 subset. The experimental design used a full factorial protocol: six feature sets paired with three regression algorithms (XGBoost, RFR, LASSO), totaling 18 feature–model configurations.

### 2.7. Statistical Analysis

To rigorously compare the predictive performance across the regression models (XGBoost, RFR, LASSO) and feature sets, statistical analyses were conducted on the aggregated metrics (i.e., the final evaluated scores across the eight distinct feature sets, *N* = 8) rather than the raw window instances.

Differences among the three models were assessed using a Repeated Measures One-Way Analysis of Variance (RM One-Way ANOVA). The Geisser–Greenhouse correction was applied to adjust for potential violations of sphericity. To control the family-wise error rate during multiple comparisons, Tukey’s post hoc test was employed, yielding adjusted *p*-values and 95% confidence intervals for the differences between models. The effect size was estimated using Eta-squared ($\eta^{2}$). Statistical significance was defined at *p* < 0.05. All statistical procedures were performed using GraphPad Prism (Version 10, GraphPad Software, Boston, MA, USA).

## 3. Results

This section presents the H3FS strategy results, followed by a comparative analysis against baselines across diverse models, joints, and teams. The robustness of the proposed evaluation index is also examined. All computations were performed using Python 3.9.19 on an NVIDIA GeForce RTX 4060 GPU (32 GB RAM).

### 3.1. Feature Selection and Evaluation

**Figure 5:**
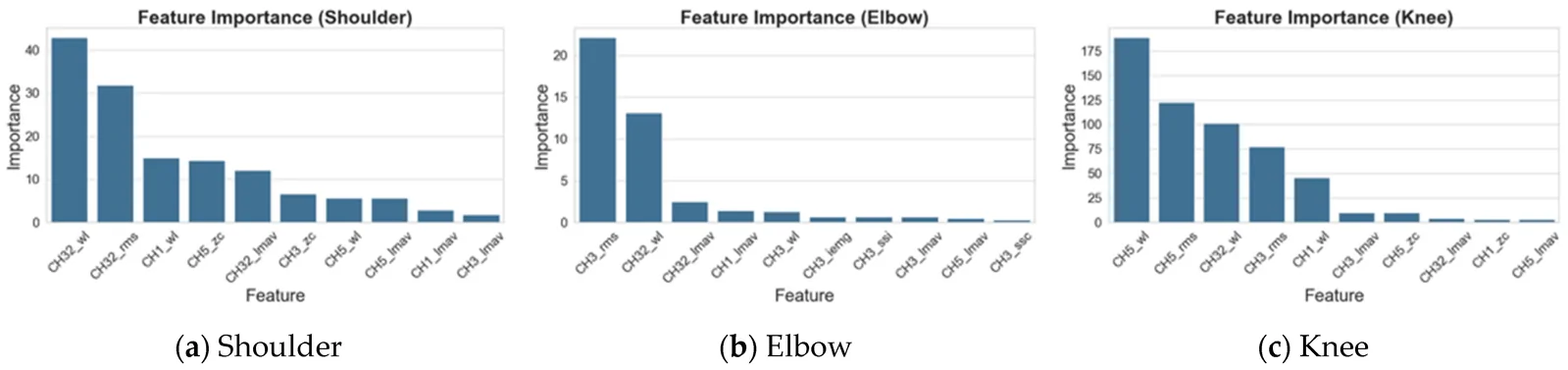
Top 10 feature importance on the LASSO model across three joints. “CHx-Feature” denotes the feature extracted from channel x. The vertical axis represents the LASSO coefficient, indicating each feature’s contribution.

The H3FS strategy was deployed to systematically evaluate the efficacy of features within the 21-dimensional candidate space. Stage 1 established performance benchmarks using the classical Hudgin set and a composite non-frequency domain baseline. In Stage 2, features were prioritized via four ranking protocols—Mutual Information, LASSO coefficients, XGBoost importance, and RFR importance—augmented by wrapper-based optimization (Ordered Forward Wrapping and Profile Likelihood Maximization). Validation across three regression architectures found the LASSO-derived subset (LMAV, WL, RMS) was optimal, showing strong correlation with joint torque output across all joints (Figure 5).

**Figure 6:**
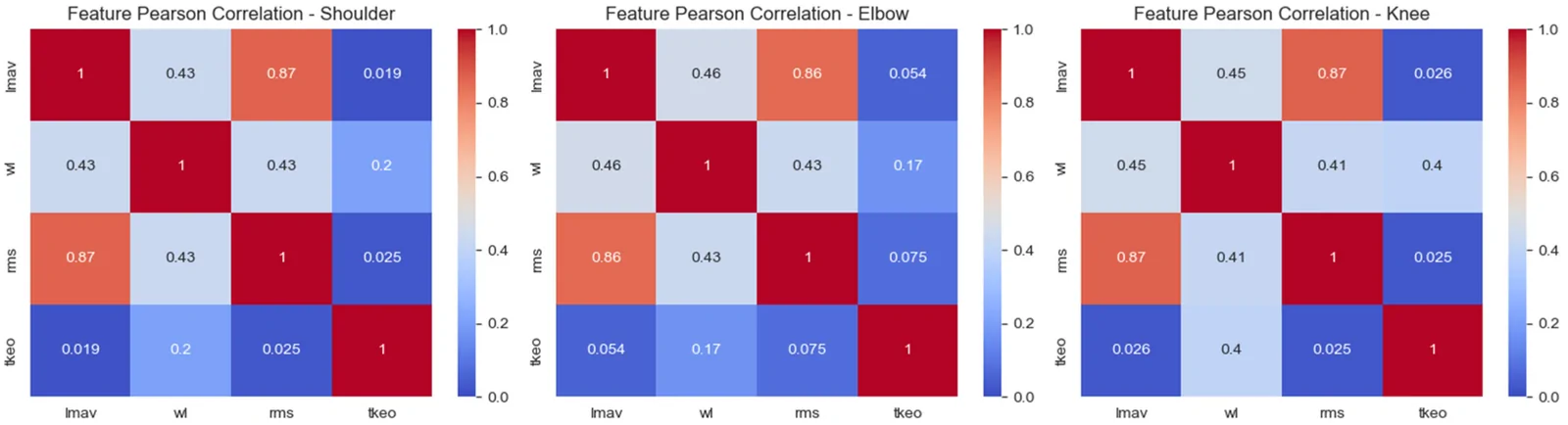
Pearson correlation matrices of the ELATE-3 features (LMAV, WL, RMS, TKEO) across the three joints.

In Stage 3, this core subset underwent structural refinement to yield the specific ELATE configuration. To improve sensitivity to transient signal energy fluctuations, the Teager–Kaiser Energy Operator (TKEO) was integrated, forming the intermediate ELATE-4 set (comprising LMAV, RMS, WL, and TKEO). However, the Pearson correlation analysis (Figure 6) revealed strong multicollinearity (r > 0.85) between RMS and LMAV, indicating significant redundancy in the information. Consequently, RMS was pruned to reduce dimensionality while preserving signal fidelity. The final configuration, ELATE-3 (comprising LMAV, WL, and TKEO), represents a minimized, highly discriminative feature set.

**Figure 7:**
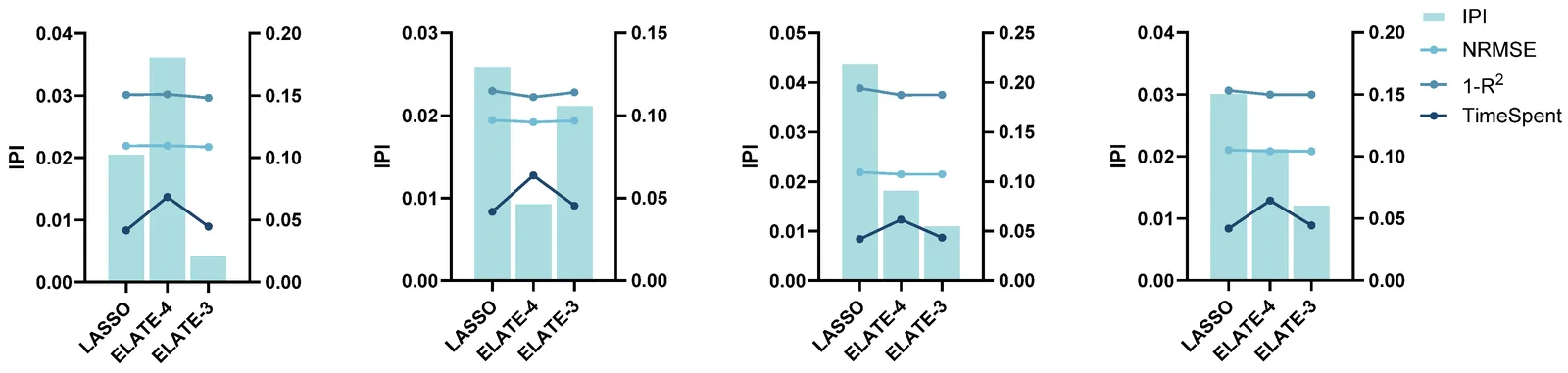
Cross-joint evaluation of feature set performance based on IPI and related metrics, demonstrating the stability of ELATE-3 across muscle groups.

To quantify the specific contributions of the constituent features, we conducted a stepwise ablation analysis (Figure 7). Incorporating TKEO into the baseline yielded measurable improvements in both NRMSE and R^2^, with only a marginal increase in computational latency, confirming its utility for capturing transient energy dynamics. Subsequently, pruning the redundant RMS feature to form the final ELATE-3 configuration significantly enhanced computational efficiency without compromising predictive fidelity. These results, quantified by the minimized IPI, validate the efficacy of the proposed strategy, demonstrating that ELATE-3 achieves a favorable balance of feature compactness, physiological interpretability, and operational efficiency.

### 3.2. Performance Comparison of Regression Models Across Feature Sets

Performance evaluation was conducted using the three regression architectures (XGBoost, RFR, LASSO) paired with the full spectrum of feature sets, including the baselines (Hudgin, FS-NFD), ranking-based subsets (FS-MI, FS-XGB, FS-RFR, FS-L), and the proposed ELATE variants. The analysis utilized a dataset collected from 39 athletes across four teams. As detailed in Section 2.1, the continuous time-series data yielded a total of 320,837 sliding windows, which were stratified by joint into the shoulder (*n* = 155,934), elbow (*n* = 119,092), and knee (*n* = 45,811) tasks. The dataset was then partitioned into training, validation, and testing sets with an approximate 7:1:2 ratio. Specifically, this partition was performed at the subject level: athletes were randomly assigned to the training (*n* = 27), validation (*n* = 4), and testing (*n* = 8) cohorts. Consequently, all sliding windows from any given subject were strictly assigned to a single subset. Model effectiveness was measured by NRMSE, R^2^, inference latency, and the IPI metric.

**Figure 8:**
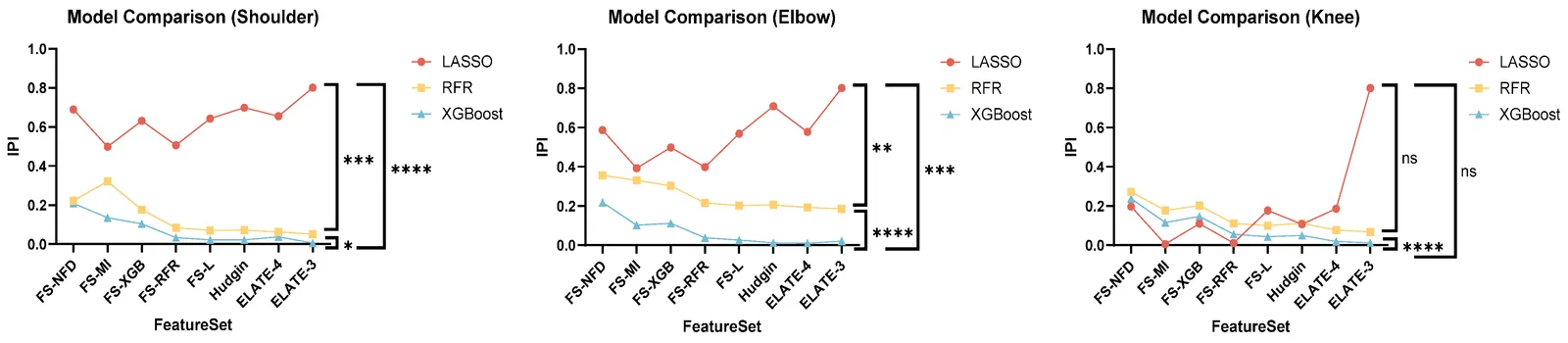
Performance comparison of LASSO, RFR, and XGBoost on multiple feature sets for three joint tasks. Asterisks indicate statistical significance between models (ns: not significant; * *p* < 0.05; ** *p* < 0.01; *** *p* < 0.001; **** *p* < 0.0001).

Figure 8 and Table 4 illustrate the comparative performance of the regression architectures. XGBoost demonstrated superior predictive fidelity across the majority of feature configurations, consistently yielding lower RMSE and higher R^2^ values than RFR and LASSO. While LASSO marginally outperformed XGBoost in specific knee-joint tasks (using FS-NFD, FS-MI, FS-XGB, and FS-RFR), the XGBoost–ELATE-3 configuration emerged as the optimal configuration. This combination achieved the lowest average RMSE across all joints (shoulder: 16.2, elbow: 6.8, knee: 25.5) and the highest determination coefficient (R^2^ = 0.85) while maintaining negligible inference latency (<0.05 ms per window). Consequently, these objective metrics independently confirm its superior balance of accuracy and real-time efficiency. In contrast, while RFR offered competitive accuracy, its higher computational overhead (0.05–0.07 ms) renders it less optimal for strict edge-computing constraints. Conversely, LASSO, despite its low computational cost, exhibited significantly higher error rates, reflecting its inherent inability to capture the nonlinear dynamics of the EMG–torque relationship.

Notably, integrating the TKEO operator into ELATE-3 significantly enhanced sensitivity to transient neuromuscular fluctuations without imposing a substantial computational penalty. While the intermediate ELATE-4 variant (augmented with RMS) was evaluated, it yielded negligible performance gains over ELATE-3. Given the high collinearity between RMS and LMAV, retaining RMS effectively increased dimensionality—and consequently the IPI—without contributing unique variance. In contrast, conventional baselines such as the Hudgin and FS-MI sets, though stable, consistently underperformed relative to the H3FS-derived model. These findings validate the core premise of the H3FS strategy: that a hierarchically screened, model-driven selection strategy outperforms static, domain-generic feature definitions.

In summary, the synergy between the XGBoost architecture and the ELATE-3 feature subset yielded the most robust performance profile among all evaluated configurations. This combination achieved superior predictive fidelity and cross-subject generalization while minimizing computational latency. These attributes establish the XGBoost–ELATE-3 strategy as a highly viable candidate, demonstrating low computational latency on the current evaluation platform and laying a technical foundation for future clinically oriented embedded real-time applications.

### 3.3. Performance Comparison Across Different Joints

To verify the H3FS strategy’s generalization, this study compared joint torque estimation at the shoulder, elbow, and knee using various regression models and features.

In the shoulder articulation task, predictive stability was consistent across nonlinear models, with R^2^ values approximating 0.85. The XGBoost–ELATE-3 configuration emerged as the optimal configuration, yielding the lowest RMSE (16.224) and a minimal IPI (0.005), thereby confirming the most favorable trade-off between accuracy and efficiency. While the RFR model matched this predictive fidelity, its elevated computational overhead resulted in a significantly penalized IPI (0.051). Conversely, the linear LASSO model exhibited marked performance degradation (RMSE > 21, R^2^ < 0.74), highlighting the necessity of nonlinear modeling to capture the complex multi-muscle synergies governing shoulder mechanics.

The elbow flexion task exhibited the highest overall predictive stability, with R^2^ values ranging from 0.86 to 0.89 and RMSE between 6.8 and 7.0. This performance ceiling suggests that the EMG–torque relationship in uniplanar upper-limb flexion is comparatively deterministic and linear. Here, XGBoost–ELATE-4 achieved marginally superior numerical accuracy (R^2^ = 0.889, RMSE = 6.829, IPI = 0.01). However, the ELATE-3 variant yielded nearly identical precision (R^2^ = 0.886) with reduced computational cost. Consequently, both configurations are viable, with ELATE-3 offering a more parsimonious feature representation and ELATE-4 providing maximal theoretical accuracy.

The knee assessment presented the greatest challenge, as evidenced by a higher baseline RMSE (25–26) and a lower R^2^ (0.81). These metrics reflect the elevated biomechanical complexity and signal variance inherent to high-force lower-limb actuation. Despite these perturbations, the XGBoost–ELATE-3 strategy demonstrated exceptional robustness, achieving the lowest RMSE (25.459) and smallest IPI (0.012). While ELATE-4 provided similar accuracy, its computational latency increased without proportional gain. Both RFR and LASSO showed significant error and instability, indicating that simple ensemble or linear baselines cannot resolve the complex nonlinear synergy patterns of the lower extremity.

To quantify the strategy’s holistic generalization capacity, we aggregated performance metrics across all joints (Table 5). The independent analysis on the test set reveals that the XGBoost–ELATE-3 configuration achieved superior aggregate performance, recording the lowest mean RMSE (16.16) and the highest R^2^ (0.85), accompanied by a sub-millisecond raw inference time (0.044 ms). These metrics significantly surpassed the established baselines, including the Hudgin set. While the intermediate ELATE-4 variant yielded marginal accuracy gains in specific subsets (e.g., the elbow), the associated computational penalty negated these benefits in the composite evaluation. This confirms ELATE-3 as the optimal configuration, providing the strongest balance between predictive accuracy and system delay.

Collectively, the consistent stability of ELATE-3 across the shoulder, elbow, and knee substantiates its ability to capture invariant neuromuscular activation signatures, independent of specific joint mechanics. These findings confirm H3FS’s strong cross-muscle and cross-task generalization, making it a robust choice for multi-joint rehabilitation and complex motor training.

### 3.4. Cross-Team Generalization Performance

To evaluate the generalization ability of the proposed ELATE-3 configuration across different athlete groups, this study adopted a cross-team validation strategy. In this specific analysis, ELATE-3 was benchmarked exclusively against the classical Hudgin feature set. In each validation run, data from three out of the four teams were used as the training set, while the remaining team was held out as the test set. The torque estimation results for each team across the shoulder, elbow, and knee joints are summarized in Table 6.

Overall, ELATE-3 outperformed the Hudgin across nearly all experimental conditions, with the exception of the elbow joint tasks for the archery and wrestling teams, where performance was marginally lower. Regarding the shoulder joint, ELATE-3 yielded distinct reductions in RMSE and increases in R^2^ in three out of the four teams, indicating enhanced stability in a joint characterized by relatively high cross-subject variability. For the elbow joint, two teams showed notable improvements in accuracy, while the remaining two teams performed comparably to the Hudgin baseline, suggesting that ELATE-3 maintains general stability at this articulation. In the knee joint tasks, ELATE-3 achieved lower RMSE and higher R^2^ values across all four teams, demonstrating the most significant improvement in generalization among the three joints. These results indicate that ELATE-3 captures the EMG–torque patterns of lower-limb muscles more effectively across diverse sporting disciplines.

In the cross-team generalization experiments, ELATE-3 demonstrated superior robustness and consistent transfer capabilities across the evaluated athletic teams, consistently outperforming the Hudgin baseline. Its performance advantage was particularly distinct in the knee joint task, a domain characterized by elevated biomechanical complexity. These findings imply that ELATE-3 demonstrates consistent performance across the tested joints and athletic disciplines, rendering it highly suitable for practical training environments subject to inter-group variability. Consequently, relative to the traditional Hudgin baseline, ELATE-3 establishes a reliable strategy for joint torque estimation across the tested athletic backgrounds and specific joint tasks.

### 3.5. Sensitivity Analysis of the IPI

To evaluate the stability of the proposed IPI across diverse weight configurations, a sensitivity analysis was conducted to quantify the influence of its constituent parameters. Multiple weight permutations satisfying the unity constraint were systematically evaluated. For each permutation, the Spearman rank correlation coefficient (*ρ*) was computed between the resulting IPI hierarchy and a designated baseline configuration (*α* = 0.4, *β* = 0.4, *γ* = 0.2). This quantitative assessment served to verify the consistency and robustness of the model rankings under varying hyperparameter conditions [35].

**Figure 9:**
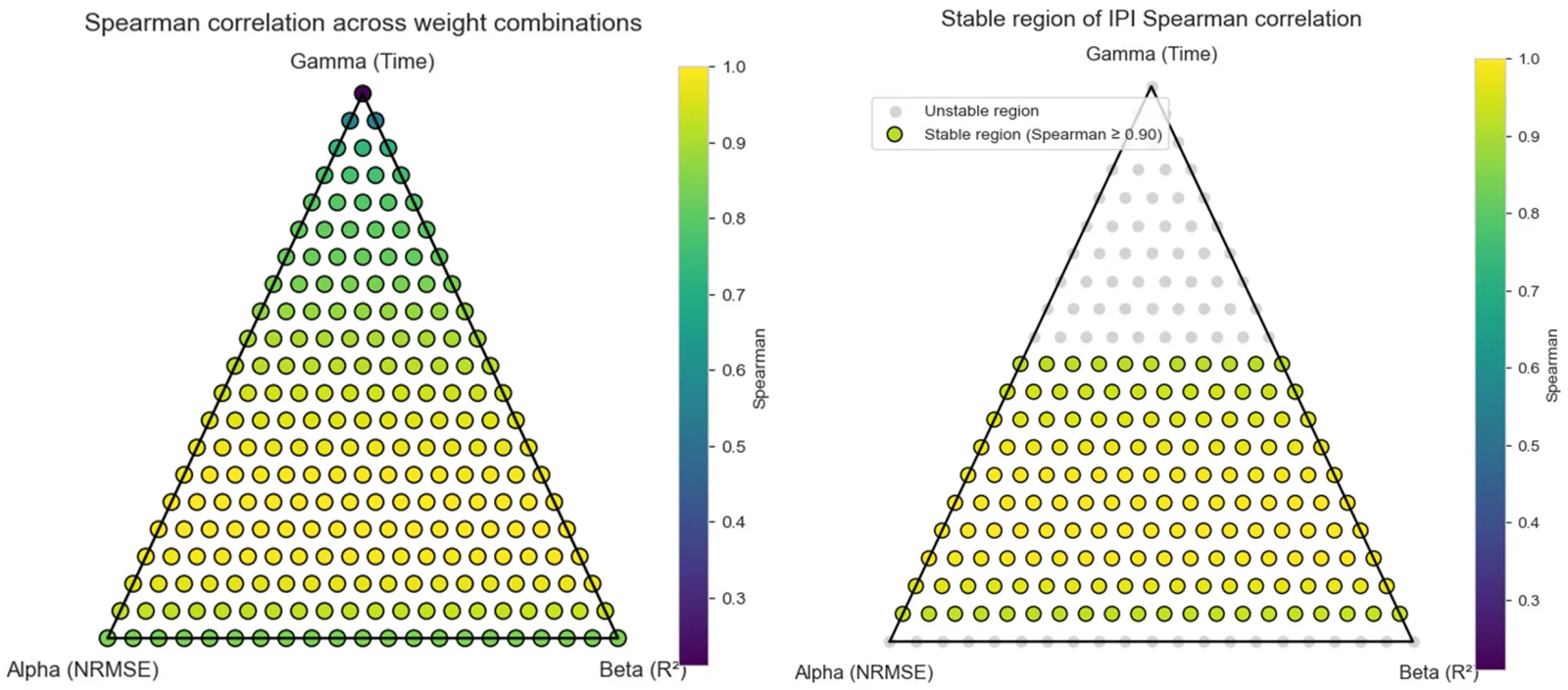
Spearman correlation distribution and stable region identification for IPI weight combinations.

As illustrated in Figure 9, the preponderance of weight configurations yielded elevated Spearman coefficients (*ρ* > 0.5), indicating that the IPI preserves a stable ranking hierarchy across diverse weighting schemes. When the accuracy-oriented coefficients (*α* and *β*) were prioritized, the resulting model rankings exhibited high consistency with the baseline configuration (*ρ* ≈ 0.9–1.0). Conversely, as the computational latency weight (*γ*) increased, the correlation exhibited a gradual decline yet remained within a moderate range. Furthermore, defining *ρ* ≥ 0.90 as the criterion for stability, Figure 9 demonstrates that the high-stability region was predominantly localized within the interval *γ* ≤ 0.35. These findings suggest that as long as computational efficiency is not overemphasized, the IPI offers strong, consistent performance evaluations across different configuration parameters, justifying its use as a reliable criterion for selecting the ELATE-3 configuration.

## 4. Discussion

Unlike traditional methods that depend on empirical choices or isolated statistical criteria, the H3FS strategy integrates domain expertise, statistical evaluation, and model-driven screening, shifting the feature selection process from a heuristic, ‘experience-driven’ approach to a rigorous, principled one. The experimental results show that the ELATE-3 feature set, created using the H3FS strategy and paired with the XGBoost regression model, achieves an ideal balance between prediction accuracy and computational efficiency across shoulder, elbow, and knee joints in various athletic groups, while also demonstrating better generalization capabilities.

### 4.1. sEMG Feature Methodological Analysis

The predictive superiority of ELATE-3 is intrinsically linked to its strong physiological interpretability. Specifically, LMAV quantifies the aggregate amplitude of the electromyographic signal, WL characterizes signal complexity and variations in neural drive, and TKEO amplifies sensitivity to instantaneous energy fluctuations [36]. This synergistic combination enables the simultaneous capture of steady-state muscle contractions and rapid dynamic transitions, thereby ensuring consistent performance across diverse kinematic profiles and subjects. Within athletic populations, chronic training induces systematic adaptations in motor unit recruitment strategies, neuromuscular coordination, and energy distribution profiles. The selected time-domain and complexity metrics exhibit high sensitivity to these physiological modifications—including amplitude modulation and shifts in high-frequency spectral content—which are often exacerbated in athletic cohorts by sport-specific training effects [37]. Consequently, these features provide a robust mechanism for monitoring joint torque dynamics in elite performers.

Regarding the current landscape of feature selection, while deep learning paradigms (e.g., CNN or LSTM) often achieve high raw accuracy, their substantial computational overhead and “black-box” nature frequently preclude real-time feedback in clinical rehabilitation [38]. In contrast, the innovation of our approach lies in identifying a “minimalist but high-fidelity” feature subset that satisfies the strict constraints of real-time deployment. By maintaining an inference latency below 0.05 ms without compromising predictive fidelity (R^2^ = 0.85), ELATE-3 offers a more pragmatic solution for embedded edge-computing platforms than high-complexity SOTA models. It should be clarified that this strategy estimates isokinetic joint torque during dynamic movement rather than static “muscle strength”, aligning with fundamental biomechanical principles.

### 4.2. ELATE-3 Feature Set Performance Analysis

A distinct dependency between feature nonlinearity and model architecture was observed: ELATE-3 excelled with XGBoost but degraded significantly under LASSO. This divergence highlights that ELATE-3’s nonlinear constituents (LMAV, WL, TKEO)—which encode high-order dynamics [39]—require the recursive partitioning of tree-based ensembles, such as XGBoost, for effective resolution. In contrast, LASSO’s linear formulation is incapable of capturing the complex feature interactions and transient variations inherent to WL and TKEO [28,29], resulting in substantial predictive attenuation when such rich temporal information is constrained by linear frameworks.

These findings confirm that predictive efficacy requires not only physiological interpretability, but also a structural match between the properties of features and the regression architecture [40]. Although the initial LASSO ranking identified LMAV, RMS, and WL, the subsequent replacement of RMS with TKEO—driven by the IPI criterion to mitigate multicollinearity and enhance sensitivity to energy transients—introduced significant nonlinear variance. This shift fundamentally violated LASSO’s strict linearity assumption (L1 regularization) [41], preventing the linear solver from capturing the complex dependencies within the enhanced feature space and leading to the observed performance degradation [42].

Furthermore, the strategy exhibited exceptional generalization across distinct articulations and athletic teams. In cross-joint evaluations, ELATE-3 maintained consistent fidelity for the shoulder, elbow, and knee, indicating adaptability to signal heterogeneity independent of joint mechanics. Similarly, cross-discipline validation demonstrated robust performance, consistently outperforming the Hudgin baseline, particularly in knee tasks. While this study did not involve longitudinal testing across multiple sessions, the exceptional cross-team generalization observed across four distinct athletic disciplines provides a strong proxy for the robustness of ELATE-3. The ability to maintain stable estimation across divergent functional profiles—ranging from stability-focused archery to power-oriented Taekwondo—suggests that ELATE-3 effectively captures consistent neuromuscular activation patterns across the tested athletic disciplines rather than over-fitting to subject-specific or session-specific noise. This inherent stability implies that the H3FS-derived features are grounded in fundamental motor control strategies, offering significant potential for long-term reliability in practical rehabilitation and motor training environments. However, the numerical improvements over classical baselines were relatively moderate and varied across joints. This non-uniformity reflects the inherent biomechanical complexities of cross-joint sEMG decoding, as differences in anatomical structures, motor unit recruitment strategies, and localized signal characteristics naturally lead to joint-specific performance variations.

### 4.3. The IPI Evaluation Perspective

The Integrated Performance Index (IPI) introduced serves as a pragmatic decision-support framework to address a common methodological limitation: the over-reliance on single, accuracy-centric metrics. In applied fields like rehabilitation and human–machine interaction, clinical utility hinges not only on predictive fidelity but also on real-time responsiveness and computational simplicity. Unlike traditional post hoc metrics, the H3FS strategy explicitly adopts the IPI as a consistent decision criterion to guide iterative feature pruning. This ensures that the transition from the candidate pool to the ELATE-3 set is governed by a reproducible metric. Sensitivity analysis validates the metric’s robustness, demonstrating consistent performance rankings across varying weight settings (specifically, γ ≤ 0.35). Although the weight configuration reflects a subjective preference, sensitivity analysis confirms high ranking stability, providing a pragmatic framework for efficiency–accuracy trade-offs.

### 4.4. Limitations and Future Directions

Despite promising results, several limitations warrant addressing. First, the full spatiotemporal potential of the 32-channel HD-sEMG array—specifically, advanced decoding techniques like spatial filtering and motor unit decomposition—was not fully exploited. Future studies will leverage these capabilities to elucidate detailed neuromuscular function. Second, aggregating data across angular velocities facilitated generalization but may obscure velocity-dependent modulation mechanisms; thus, future research will investigate velocity-specific feature selection to resolve these kinematic dependencies. Third, although MVC-based normalization is standard, this study used peak-torque-based normalization due to logistical constraints in testing high-performance athletes across diverse environments. We acknowledge that the lack of a standardized MVIC protocol may affect cross-subject comparability, and integrating online adaptive calibration algorithms to minimize this overhead remains a priority. Fourth, the IPI relies on min–max normalization, rendering it a relative ranking metric specific to the evaluated model cohort rather than an absolute cross-study benchmark; future iterations will explore absolute threshold-based normalizations to yield globally comparable indices. Finally, to improve clinical extrapolation, future initiatives will expand the data repository, integrate deep representation learning, and rigorously evaluate robustness under dynamic fatigue, with the ultimate goal of deploying the strategy on embedded edge-computing platforms for real-time rehabilitation [39]. Crucially, as the current study was validated exclusively on healthy young athletes without neurological or musculoskeletal pathologies, its clinical validity remains to be established. Translation into clinical practice requires separate, independent validation in targeted patient populations, meticulously accounting for pathological neuromuscular activity and altered biomechanics.

## 5. Conclusions

This study introduces the Hierarchical Three-Stage Feature Selection (H3FS) strategy, which synergizes domain knowledge with statistical and model-driven screening to identify a compact, interpretable feature set, ELATE-3 (LMAV, WL, TKEO). Paired with XGBoost, ELATE-3 achieved superior predictive accuracy and negligible latency across the shoulder, elbow, and knee joints. We also established the Integrated Performance Index (IPI) to quantify the trade-off between accuracy and efficiency, thereby verifying the strategy’s low computational latency on the adopted platform and its potential for future real-time implementations. Results highlight the critical synergy between feature nonlinearity and model architecture, while robust performance across diverse athletic teams confirms the strategy’s transferability. Future work will focus on evaluating stability under fatigue and dynamic kinematics and implementing the system on embedded hardware for practical deployment, alongside targeted validations for potential rehabilitation monitoring.

## Figures and Tables

**Table 1:** Joint-specific testing posture and EMG electrode placement during the isokinetic tasks.

Joint	Subject Posture	Electrode Placement	Motion Range
Shoulder	Seated, pelvis/trunk stabilized. Arm extended, holding the lever. Shoulder axis aligned with dynamometer axis.	Deltoid, Biceps Brachii, Brachioradialis, Triceps Brachii	0° → 180° → 0°
Elbow	Seated, pelvis/trunk stabilized. Upper arm supported and strapped. The elbow axis is aligned with the dynamometer axis.	Deltoid, Biceps Brachii, Brachioradialis, Triceps Brachii	0° → 135° → 0°
Knee	Seated, pelvis/trunk stabilized. The lower leg is in padded support and strapped. Knee axis aligned with dynamometer axis.	Quadriceps, Gastrocnemius, Tibialis Anterior, Biceps Femoris	0° → 90° → 0°

In the electrode placement column, the first three muscles listed were acquired using standard distributed electrodes, whereas the final muscle in each list was recorded using the 32-channel flexible electrode array.

**Table 2:** Definitions of extracted sEMG features.

	Feature	Abbreviation	Mathematical Definitions
1	Integrated EMG	IEMG	$IEMG=\sum_{i=1}^{N}\left|x_{i}\right|$
2	Mean Absolute Value	MAV	$MAV=\frac{1}{N}\sum_{i=1}^{N}\left|x_{i}\right|$
3	Log Mean Absolute Value	LMAV	$LMAV=\ln\left(MAV+10^{-12}\right)$
4	Simple Square Integral	SSI	$SSI=\sum_{i=1}^{N}x_{i}^{2}$
5	Root Mean Square	RMS	$RMS=\sqrt{\frac{1}{N}\sum_{i=1}^{N}x_{i}^{2}}$
6	Variance	VAR	$VAR=\frac{1}{N}\sum_{i=1}^{N}{\left(x_{i}-\bar{x}\right)}^{2}$
7	Standard Deviation	SD	$SD=\sqrt{VAR}$
8	Waveform Length	WL	$WL=\sum_{i=1}^{N-1}\left|x_{i+1}-x_{i}\right|$
9	Slope Sign Changes	SSC	$SSC=\sum_{i=2}^{N-1}1[\left(x_{i}-x_{i-1}\right)\left(x_{i}-x_{i+1}\right)<0]$
10	Willison Amplitude	WAMP	$WAMP=\sum_{i=1}^{N-1}1[|x_{i+1}-x_{i}|>\theta ]$
11	Zero Crossing	ZC	$ZC=\sum_{i=1}^{N-1}1[x_{i+1}x_{i}<0]$
12	V-Order	V	$V=\frac{1}{N}\sum_{i=2}^{N-1}1[\left(x_{i}>x_{i-1}\right)\wedge \left(x_{i}>x_{i+1}\right)]$
13	Total Power	TP	TP=∑P(f)
14	Median Power	MP	MP=median(P(f))
15	Median Frequency	MDF	$\int_{0}^{MDF}P\left(f\right)df=\frac{1}{2}\int_{0}^{f_{max}}P\left(f\right)df$
16	Mean Frequency	MNF	$MNF=\frac{\sum f_{i}P(f_{i})}{\sum P(f_{i})}$
17	Power Spectrum	PS	$PS=\frac{1}{M}\sum P(f_{i})$
18	Fuzzy Entropy	FE	FE=FuzzyEn(x,m,r)
19	Hjorth Parameter Mobility	HPM	$HPM=\sqrt{\frac{Var(\dot{x})}{Var\left(x\right)}}$
20	Hjorth Parameter Complexity	HPC	$HPC=\frac{HPM(\dot{x})}{HPM\left(x\right)}$
21	Normalized Shape Variation	NSV	$NSV=\frac{1}{N}\sum_{i=1}^{N}{\left(\frac{x_{i}-\bar{x}}{SD}\right)}^{4}$

**Table 3:** Baseline feature sets.

Feature Set	Abbreviation	Features
Hudgin feature set	Hudgin	MAV, WL, SSC, ZC
Non-frequency-domain feature set	FS-NFD	IEMG, MAV, LMAV, SSI, RMS, VAR, SD, WL, SSC, WAMP, V, HPM, HPC, NSV, ZC
Mutual information ranking feature set	FS-MI	IEMG, MAV, LMAV, SSI, RMS
XGBoost-based feature importance ranking feature set	FS-XGB	IEMG, SSI, WL, TP
RFR-based feature importance ranking feature set	FS-RFR	IEMG, MAV, LMAV, SSI, RMS, WL
LASSO-based feature importance ranking feature set	FS-L	LMAV, RMS, WL

**Table 4:** Comparison of three-joint torque estimation across regression models and feature sets.

Regression Model	Feature Set	RMSE	R^2^	TimeSpent	IPI
XGBoost	FS-NFD	16.272/6.824/25.744	0.851/0.886/0.807	0.479/0.499/0.494	0.209/0.219/0.238
	FS-MI	17.086/7.167/26.745	0.835/0.877/0.794	0.054/0.055/0.054	0.135/0.102/0.115
	FS-XGB	16.229/6.796/25.856	0.852/0.889/0.805	0.272/0.272/0.273	0.104/0.112/0.147
	FS-RFR	16.387/6.836/25.821	0.849/0.885/0.806	0.068/0.068/0.068	0.033/0.038/0.056
	FS-L	16.387/6.836/25.821	0.849/0.885/0.806	0.042/0.042/0.042	0.021/0.026/0.044
	Hudgin	16.324/6.752/25.865	0.850/0.888/0.805	0.057/0.057/0.057	0.021/0.012/0.050
	ELATE-4	16.403/6.829/25.470	0.849/0.889/0.813	0.068/0.064/0.062	0.037/0.010/0.019
	ELATE-3	16.224/6.807/25.459	0.852/0.886/0.812	0.045/0.045/0.043	0.005/0.021/0.012
RFR	FS-NFD	16.321/7.606/26.157	0.850/0.870/0.801	0.485/0.505/0.504	0.222/0.356/0.273
	FS-MI	18.240/8.179/27.380	0.810/0.848/0.783	0.060/0.061/0.064	0.321/0.331/0.178
	FS-XGB	16.623/7.779/26.452	0.844/0.864/0.796	0.277/0.278/0.284	0.176/0.303/0.202
	FS-RFR	16.620/7.777/26.422	0.844/0.865/0.797	0.074/0.076/0.080	0.084/0.215/0.111
	FS-L	16.607/7.775/26.438	0.844/0.865/0.797	0.048/0.048/0.053	0.070/0.202/0.100
	Hudgin	16.567/7.765/26.476	0.845/0.865/0.796	0.063/0.065/0.071	0.071/0.206/0.112
	ELATE-4	16.468/7.722/26.001	0.847/0.867/0.802	0.075/0.071/0.075	0.062/0.193/0.077
	ELATE-3	16.471/7.732/26.012	0.846/0.867/0.802	0.050/0.051/0.053	0.051/0.186/0.068
LASSO	FS-NFD	19.684/7.813/25.368	0.783/0.836/0.814	0.479/0.498/0.494	0.690/0.587/0.197
	FS-MI	19.691/7.808/25.341	0.783/0.836/0.814	0.054/0.055/0.054	0.499/0.393/0.005
	FS-XGB	19.934/7.850/25.465	0.778/0.834/0.812	0.272/0.272/0.273	0.631/0.499/0.110
	FS-RFR	19.691/7.808/25.341	0.783/0.836/0.814	0.068/0.068/0.068	0.506/0.399/0.011
	FS-L	20.444/8.210/27.605	0.761/0.808/0.776	0.042/0.042/0.042	0.643/0.569/0.177
	Hudgin	20.709/8.550/26.640	0.754/0.789/0.794	0.057/0.057/0.057	0.699/0.709/0.107
	ELATE-4	20.444/8.210/27.605	0.761/0.808/0.776	0.068/0.064/0.062	0.655/0.578/0.186
	ELATE-3	21.472/9.059/34.936	0.737/0.775/0.635	0.045/0.045/0.043	0.801/0.802/0.801

Values are reported in the order of shoulder/elbow/knee.

**Table 5:** Comparison of XGBoost’s estimation results of joint torque in different feature sets.

Feature Set	Joint	RMSE	R^2^	TimeSpent	IPI
FS-NFD	Shoulder	16.272	0.851	0.479	0.209
	Elbow	6.824	0.886	0.499	0.219
	Knee	25.744	0.807	0.494	0.238
	Mean	16.280	0.848	0.491	0.222
FS-L	Shoulder	16.387	0.849	0.042	0.021
	Elbow	6.836	0.885	0.042	0.026
	Knee	25.821	0.806	0.042	0.044
	Mean	16.348	0.847	0.042	0.030
Hudgin	Shoulder	16.324	0.850	0.057	0.021
	Elbow	6.752	0.888	0.057	0.012
	Knee	25.865	0.805	0.057	0.050
	Mean	16.314	0.848	0.057	0.028
ELATE-4	Shoulder	16.403	0.849	0.068	0.037
	Elbow	6.829	0.889	0.064	0.010
	Knee	25.470	0.813	0.062	0.019
	Mean	16.234	0.850	0.065	0.022
ELATE-3	Shoulder	16.224	0.852	0.045	0.005
	Elbow	6.807	0.886	0.045	0.021
	Knee	25.459	0.812	0.043	0.012
	Mean	16.163	0.850	0.044	0.013

**Table 6:** Cross-team generalization performance of ELATE-3 compared with the Hudgin feature set.

Team	Joint	Hudgin RMSE	ELATE-3 RMSE	Hudgin R^2^	ELATE-3 R^2^
Archery	Shoulder	16.199	15.926	0.814	0.820
	Elbow	7.726	7.937	0.833	0.823
	Knee	30.069	27.555	0.798	0.822
Wrestling	Shoulder	14.158	13.659	0.859	0.868
	Elbow	6.432	6.521	0.896	0.895
	Knee	27.160	27.137	0.762	0.765
Rock Climbing	Shoulder	12.373	12.407	0.591	0.593
	Elbow	6.615	6.496	0.798	0.802
	Knee	26.520	25.523	0.859	0.868
Taekwondo	Shoulder	13.989	13.849	0.584	0.593
	Elbow	6.459	6.277	0.879	0.886
	Knee	28.287	26.304	0.803	0.820

## Data Availability

The data are not publicly available due to privacy and ethical restrictions involving human participants.

## Abbreviations

The following abbreviations are used in this manuscript:

sEMG	Surface Electromyography
MVC	Maximum Voluntary Contraction
H3FS	Hierarchical Three-Stage Feature Selection
IPI	Integrated Performance Index
ELATE	EMG LASSO and Teager–Kaiser Enhanced
LMAV	Logarithmic Mean Absolute Value
WL	Waveform Length
TKEO	Teager–Kaiser Energy Operator
RMS	Root Mean Square
